# Validation of a GPU‐Based 3D dose calculator for modulated beams

**DOI:** 10.1002/acm2.12074

**Published:** 2017-03-29

**Authors:** Saeed Ahmed, Dylan Hunt, Jeff Kapatoes, Robert Hayward, Geoffrey Zhang, Eduardo G. Moros, Vladimir Feygelman

**Affiliations:** ^1^ Departement of Physics University of South Florida Tampa FL USA; ^2^ Departement of Radiation Oncology Moffitt Cancer Center Tampa FL USA; ^3^ Sun Nuclear Corp. Melbourne FL USA

**Keywords:** convolution/superposition, dose calculations, GPU‐accelerated calculations, segmented beams

## Abstract

A superposition/convolution GPU‐accelerated dose computation algorithm (the Calculator) has been recently incorporated into commercial software. The algorithm requires validation prior to clinical use. Three photon energies were examined: conventional 6 MV and 15 MV, and 10 MV flattening filter free (10 MVFFF). For a set of IMRT and VMAT plans based on four of the five AAPM Practice Guideline 5a downloadable datasets, ion chamber (IC) measurements were performed on the water‐equivalent phantoms. The average difference between the Calculator and IC was −0.3 ± 0.8% (1SD). The same plans were projected on a phantom containing a biplanar diode array. We used the forthcoming criteria for routine gamma analysis, 3% dose**–**error (global (G) normalization, 2 mm distance to agreement, and 10% low dose cutoff). The *γ* (3%G/2 mm) average passing rate was 98.9 ± 2.1%. Measurement‐guided three‐dimensional dose reconstruction on the patient CT dataset (excluding the Lung) resulted in a similar average agreement rate with the Calculator: 98.2 ± 2.0%. The mean *γ* (3%G/2 mm) passing rate comparing the Calculator to the TPS (again excluding the Lung) was 99.0 ± 1.0%. Because of the significant inhomogeneity, the Lung case was investigated separately. The calculator has an alternate heterogeneity correction mode that can change the results in the thorax for higher‐energy beams (15 MV). As this correction is nonphysical and was optimized for simple slab geometries, its application leads to mixed results when compared to the TPS and independent Monte Carlo calculations, depending on the CT dataset and the plan. The Calculator vs. TPS 15 MV Guideline 5a IMRT and VMAT plans demonstrate 96.3% and 93.4% *γ* (3%G/2 mm) passing rates respectively. For the lower energies, which should be predominantly used in the thoracic region, the passing rates for the same plans and criteria range from 98.6 to 100%. Overall, the Calculator accuracy is sufficient for the intended use.

## 1 Introduction

It is the current standard of practice in the United States that for each radiotherapy treatment course involving intensity modulated, inversely planned dose delivery (IMRT/VMAT), a patient‐specific quality assurance procedure has to be performed to ensure that the calculated dose distribution is reasonably accurate.^1^ A simple point dose verification is considered sufficient in conventional (forward‐planning) therapy.^2^ Acknowledging the complexity and temporal nature of the dose calculation and delivery of modulated beams, dose comparison has to be more extensive. Historically, patient specific IMRT QA was performed by projecting the treatment plan on a phantom containing a dosimeter and comparing the measured sample of the 3D dose distribution with calculations.^3^ As the inversely planned techniques matured and became the mainstay of radiotherapy, alternative dose verification techniques started to be actively explored. Those included electronic portal imaging device (EPID)‐based dosimetry,^4^, ^5^ calculation‐based reconstruction from the accelerator log files,^6^, ^7^ including harvesting aperture shapes (but not fluence) from the EPID,^8^ or just a straightforward recalculation by an independent dose engine.^9^, ^10^, ^11^, ^12^, ^13^ Each method has its advantages and disadvantages, and none is capable of catching every possible mode of failure,^10^ including catastrophic events.

In this article, we critically examine and validate a fast, independent 3D dose calculator as an additional tool that potentially could be incorporated in the IMRT/VMAT QA process. This calculator is the dose engine used in the commercial products (Sun Nuclear Corp., Melbourne, FL, USA) for purely calculational (DoseCHECK) or empirically guided (PerFRACTION) dose reconstruction. For the former, the DICOM RT Plan from the primary treatment planning system (TPS) serves as the input for the verification dose calculation on the patient CT dataset. In the latter, the aperture shapes recorded by the EPID during beam delivery are used to generate the MLC control points. Simultaneously, corresponding monitor unit and angle progressions are harvested from the accelerator log files for dose calculation. A common step to both the calculational and the semi‐empirical approaches is dose calculation by a Superposition/Convolution (S/C) technique. Obviously, the accuracy of the dose calculation engine employed in these products is of paramount importance for their clinical performance and deserves a thorough investigation. Experimental validation of the algorithm is the only goal of this manuscript. The data from this manuscript was used in the Sun Nuclear Corp. White Paper.^14^ We do not attempt to weigh in on an ongoing discussion^15^, ^16^ of the relative merits of measurement‐based vs. software‐based patient‐specific IMRT QA methods.

## 2 Methods

### 2.A Calculator description

#### 2.A.1 Dose calculation algorithm

The approach was originally developed and described in detail by Jacques et al.^17^ It is a variant of a S/C style dose calculation^18^, ^19^, ^20^ adapted for fast execution on the graphics processing unit (GPU) card(s). As with all implementations of S/C methods, the calculation consists of three steps: fluence calculation, total energy released per unit mass (TERMA) calculation, and, finally, superposition.

The fluence calculation is responsible for simulating radiation transport within the linear accelerator treatment head. There are separate sources used to model primary, extra focal (scatter), and electron contamination radiation. The primary and extrafocal sources each have their own spectrum and arbitrary radial intensity profile. Jaws and MLC characteristics, including properties such as MLC tongue‐and‐groove thickness and MLC leaf end curvature, are accounted for explicitly.

In the next step, fluence is transported through the patient to compute primary energy released in the volume (TERMA). The material composition of the patient volume is determined using the CT dataset along with a user‐provided CT‐to‐relative electron density (ED) conversion table. From relative electron density, other material properties are computed by interpolation among nine predefined materials, one of which is water. The TERMA calculation uses energy‐dependent (16 bins) mass attenuation coefficients for the appropriate material,^20^ while electron density is used to attenuate the primary fluence through the patient along the heterogeneity‐corrected ray‐trace path. TERMA is calculated every 2° for VMAT plans.

Finally, the superposition step spreads the TERMA by the energy deposition kernel to determine the final dose at each point.^19^ The kernel is derived from high resolution (1 mm, 1°) Monte Carlo (MC) simulations in the water phantom. A cumulative‐cumulative kernel is used to minimize voxelization effects.^18^ The energy deposition kernel scales with electron density, which differentiates the superposition approach from the traditional convolution.^17^ The kernel's angular dependence is discretized using a collapsed cone approximation,^21^ and both kernel tilting and beam hardening are accounted for. The superposition calculation for VMAT is performed every 5°, which still allows for acceptable accuracy of the overall calculation.^17^ As TERMA calculations are substantially faster than superposition calculations, using a high TERMA but low superposition angular resolutions increases the speed of VMAT calculations.^17^

#### 2.A.2 Input data

The Calculator comes preconfigured with standard beam models for each machine class. For example, the energy spectra/beam profiles for the TrueBeam linac used in this work (Varian Medical Systems, Palo Alto, CA, USA) are based on average measurements for five machines of the same class. The same is true for the majority of relative output factors (S_cp_). However, for the small fields (≤ 4 cm) the output factors from Kerns et al.^22^ were used. The user has to supply the local CT number‐to‐ED conversion table and the absolute output per monitor unit under the reference conditions. For patient‐specific data, a set of DICOM CT and corresponding DICOM RT Plan and DICOM RT Structure objects are required. The structures determine the extent of the dose reporting volume. Also, if the density override information is present in the Structure object, it is used in dose calculations.

### 2.B Validation

The main goal of this paper is independent validation of the just described algorithm. To cover a reasonably wide range of energies and different beam types, three photon energies were examined: conventional 6 MV and 15 MV, and 10 MV flattening filter free (10 MVFFF). The validation was performed in a deliberate fashion, gradually increasing the complexity of the tests in terms of radiation fields (from basic shapes to IMRT/VMAT), analysis dimensionality (from point doses to 2D to 3D dose sampling), and dataset heterogeneity (from homogeneous phantoms to thoracic CTs).The strategy included a number of steps similar to commissioning of a primary TPS,^23^ albeit abbreviated:

1. Dose distributions for simple square fields were compared on the water phantom between the Calculator and the primary TPS (Pinnacle v. 9.8, Philips Medical Systems, Fitchburg, WI);
2. Central axis percentage depth doses (PDD) were compared on a lung‐simulating slab phantom between Pinnacle, the Calculator, and Monte Carlo (MC) simulations;
3. IMRT/VMAT plans generated on the AAPM Practice Guideline 5a^23^ datasets were recalculated on the phantoms. Point doses were measured with an ion chamber in the phantoms and compared to the doses predicted by the Calculator;
4. The Calculator dose distributions for selected plans on a homogeneous cylindrical phantom were compared against a biplanar diode array (Delta4) measurements for a limited number of points (the diodes’ locations);
5. The 3D Calculator dose on the patients’ CTs was compared to the 3D measurement‐guided dose reconstruction on the same datasets;
6. Finally, all calculated 3D patient dose distributions were compared between the Calculator and the primary TPS, as would ultimately be done in the clinic.

#### 2.B.1 Basic beams on the water phantom

The dose distributions on the synthetic CT phantom of unit density were compared to the primary TPS for a series of open square fields (5 × 5, 10 × 10, and 20 × 20 cm^2^) and a 3 × 3 cm^2^ MLC‐defined aperture in the middle of a 10 × 10 cm^2^ jaw opening. The Pinnacle Collapsed Cone Convolution^24^, ^25^ beam model in our system generally agrees with water scans for basic fields to within 1%, and thus the TPS was used as the reference to facilitate easy full 3D dose comparison. Also, it is useful to compare the basic beam data with Pinnacle, as ultimately the modulated dose distributions are compared to it as well. The absolute point dose under the TPS reference conditions, as generated by the Calculator, was found to be within 0.2% of the expected (input) dose, thus satisfying the Guideline 5a^23^ recommendations. To achieve similar agreement in Pinnacle, the calculation mode had to be switched to the homogeneous water phantom. Otherwise, a synthetic CT phantom of unit density is treated in Pinnacle as being slightly different from water, due to the CT to material assignment method.^26^ After proper reference dose was confirmed, the Pinnacle and Calculator dose grids (2 mm voxel size) were loaded in 3DVH software v. 3.3 (Sun Nuclear Corp) and compared using gamma analysis using 2% (global normalization) dose**–**error and 2 mm distance to agreement criteria, with low‐dose cut‐off at 10% of the maximum (2%G/2 mm/10%).

#### 2.B.2 Slab inhomogeneities

The calculator has two modes for handling significant inhomogeneities (e.g., lung), requiring two separate beam models. The basic S/C approach was described above. Alternatively, an additional correction known as Heterogeneity‐Compensated Superposition (HCS) 27 can be applied. It relies on the patient density near the material interfaces being modified (filtered) in a position and direction sensitive manner, allowing the dose to be changed compared to the standard S/C approach. Application of this correction approximately doubles the calculation time.

Depth‐dose curves were extracted from Pinnacle, standard Calculator, HCS Calculator, and Monte Carlo (MC) calculations for 2 × 2, 5 × 5, and 10 × 10 cm^2^ fields on a wide slab phantom consisting of 5 cm of water, followed by 5 cm of lung (0.3 g/cm^3^), and 20 cm of water. All PDDs were normalized beyond their respective d_max_, at a 2 to 3 cm depth, depending on the beam energy.

Monte Carlo calculations were performed with PRIMO, a radiotherapy graphical interface to PENELOPE code.^28^ Manufacturer‐provided IAEA‐compliant phase space files for the TrueBeam accelerator^29^ were used in lieu of modeling the accelerator head above the movable jaws. The current version of those files provides a phase space on a horizontal plane 27 cm downstream from the source. The number of histories was sufficient to achieve 1% statistical uncertainty (two standard deviations) at the dose level above 50% of the maximum in the phantom. An in‐house script was written to convert PRIMO ASCII dose files into DICOM RT‐compliant dose objects. The PRIMO simulations were validated against Pinnacle for the 10 × 10 cm^2^ fields on the water phantom, at the 2%/2 mm level.

#### 2.B.3 IMRT/VMAT Planning and delivery

All measurements were done on a TrueBeam v 2.0 linear accelerator equipped with a 120‐leaf Millennium MLC (Varian Medical Systems).

The IMRT and VMAT plans were developed based on the Guideline 5a Report library of test plans.^23^ They included four realistic plans from the available downloadable datasets: Anal, Head and Neck (H&N), Abdomen and Lung. The concept behind these Guideline 5a cases is to provide challenging but clinically relevant goals, with large targets and tight constraints, resulting in highly modulated plans pushing the accuracy limits of the TPS calculation algorithms. They were previously used for large, inter‐institutional plan studies similar to the pilot study described by Nelms et al.^30^

Both VMAT (Pinnacle SmartArc^31^, ^32^) and static gantry, segmented IMRT (Pinnacle Direct Machine Parameters Optimization^33^) plans were created for each case. Optimization was done for the 6 MV beams, and other energies were simply recalculated for the same control points (CP). The H&N and Anal VMAT plans used two arcs, while the remaining plans used one. The VMAT plans were calculated with 2° angular CP increment. The IMRT plans used seven to nine equidistant gantry angles. Three of four plans (except the Abdomen), had targets too large to be encompassed for conventional IMRT with a single set of the MLC carriage positions, due to the limitations of the maximum leaf extension. They were instead planned with the “wide‐field” IMRT technique, where the leaves are allowed to nearly close inside the treatment field, not necessarily under the X jaws, but those leaf abutment points move across the field from segment to segment to avoid excessive exposure at any one location in the patient. In all cases, a uniform 2.5 mm dose grid resolution was used for both the TPS and the Calculator.

#### 2.B.4 The Calculator vs. point dose measurements

For the three of four cases (excluding the Lung), the plans were projected on the homogeneous 20 × 20 × 20 cm^3^ Plastic Water Cube phantom (CIRS Inc., Norfolk, VA, USA). Point doses in the high‐dose, low‐gradient regions were measured with a 0.125 cm^3^ Model TN31010 (PTW, Freiburg, Germany) ion chamber (IC). The chamber was cross‐calibrated in a 10 × 10 cm^2^ field against the expected primary TPS dose prior to every measurement session. The chamber volume was drawn as a region of interest and the corresponding mean dose was used for comparisons.

Unlike the previous three plans, the Lung plan was recalculated on a heterogeneous anthropomorphic Thorax Phantom (modified Model 002LFC; CIRS Inc.),^34^ with the isocenter paced at the middle of the spherical target. The phantom is based on a Plastic Water cylinder with an approximately elliptical cross‐section. The overall dimensions are 30 cm × 30 cm × 20 cm. The phantom contains two cylindrical “lungs” made of epoxy resin 0.21 g/cm^3^ in density. The right lung can accommodate a 4 cm diameter spherical Plastic Water target. The target and a number of other locations in the phantom accept an A1SL 0.05 cm^3^ IC (Standard Imaging Inc., Middleton, WI, USA). The IC measurements were performed at two points: in the target sphere and in the “mediastinum”, the latter representing a point well within the homogeneous portion of the phantom.

#### 2.B.5 The Calculator vs. biplanar diode array (homogeneous phantom)

In the next step, the Calculator dose distributions were compared to the Delta^4^ (ScandiDos AB, Uppsala, Sweden) measurements, sampling the dose volume with two orthogonal detector planes. The daily correction factor was determined by irradiating two parallel‐opposed 10 × 10 cm^2^ fields and minimizing the difference with the primary TPS in the central portion of the irradiated area. Gamma analysis with 3%G/2 mm/10% criteria was used as the primary metric here and later in this study. Results with 2%G/2 mm criteria are also presented for comparison, as warranted. Gamma analysis was performed using the Delta^4^ software, with the Calculator DICOM RT dose grid loaded as reference dose.

#### 2.B.6 The Calculator vs. 3D measurement‐guided dose reconstruction on patient CT

Following the measurements with the ArcCHECK (AC) dosimeter (Sun Nuclear Corp.), measurement‐guided dose reconstruction on the patients’ datasets was performed, using the AC‐based planned dose perturbation (ACPDP) method.^34^, ^35^, ^36^, ^37^ The AC measurements and the primary TPS (Pinnacle) dose grid were used as the required ACPDP inputs. The ACPDP dose was loaded as the reference in 3DVH software,^37^ and the Calculator dose as the comparison. Both Pinnacle and the Calculator used the CT number‐to‐density conversion tables derived from the same phantom scan. However, the physical density values were specified for Pinnacle and electron density for the Calculator. The ACPDP method generates a 3D dose grid on a patient dataset. However, it has no knowledge of the (variable) density of the patient and relies on the primary TPS to account for inhomogeneities. For the test plans excluding the Lung dataset, the density variations do not pose a problem beyond possible minor discrepancies. However, with the large low density heterogeneities, there is no guarantee that the different dose calculation algorithms would agree. Therefore, the Lung case was excluded from the ACPDP analysis and was investigated separately as described in the following sections.

The same 3%G/2 mm gamma analysis criteria were used, supplemented by 2%G/2 mm data. Since the discrepancies in the buildup region are expected, and are effectively ignored in the traditional phantom measurements by the virtue of the active volume placement at depth, the analyzed volumes here and in the next section were filtered to exclude the outermost 7 mm of the body on the CT datasets.

#### 2.B.7 The Calculator vs. primary TPS on the patient CT (Not including Lung)

The 3D Calculator doses on the patients’ datasets were directly compared to the corresponding Pinnacle dose distributions using the same methodology as described above. This configuration represents the intended use of the Calculator. The Lung case is not included in this comparison, to isolate differences in accounting for heterogeneities.

#### 2.B.8 The Calculator vs. primary TPS (Lung)

Additional comparisons were made for the Lung plan to better understand the differences, and their practical significance, between the two versions of the Calculator heterogeneity corrections. Unlike with the previous three datasets, special tests were done for the Lung plan. In addition to comparisons on the patient dataset, the plans were recalculated on the Thorax phantom with Pinnacle, no‐HCS, and HCS versions of the Calculator. The phantom provides clear‐cut interfaces and uniform low‐density regions, which were expected to emphasize the differences between various algorithms.

Comparisons with MC were also performed. Unfortunately, it is not practical to recalculate segmented beams with PRIMO. Instead, we created a simple 5‐beam coplanar plan which could be calculated on the Thorax phantom with identical parameters with every S/C algorithm and MC. All beams were equally weighted and the MLC apertures surrounded the 4 cm diameter spherical target with a 0.7 cm margin. The relative MC calculations were normalized to the isocenter dose measured with an IC. The resulting dose grids were compared in 3DVH software.

## 3 Results

### 3.A Basic beams on the water phantom

**Figure 1:**
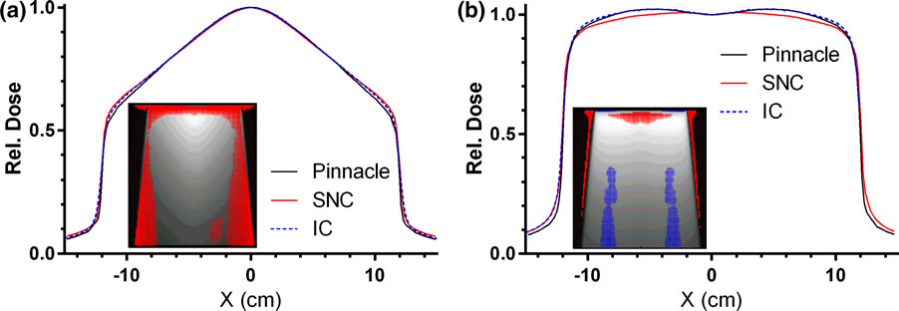
Gamma analysis (2%L/2 mm) error maps (inserts) between Pinnacle and SNC calculator, and normalized cross‐plane dose profiles at 20 cm depth for 10MVFFF (a) and 15 MV (b) 20 × 20 cm^2^ fields. Ion chamber (IC) profiles are also included for comparison. Red and blue pixels are where the Calculator dose is above and below Pinnacle respectively.

For all beams and energies the average *γ* (2%G/2 mm) agreement beyond d_max_ was 99.3 ± 1.3% (1 SD). The only plans having < 100% agreement were the 20 × 20 cm^2^ fields for 10 MVFFF and 15 MV (96.7 and 97.9%, respectively) and the MLC‐defined 3 × 3 cm^2^ 15 MV field. To emphasize the areas of disagreement for the 20 × 20 cm^2^ fields, the error maps in Fig. 1 are based on the more sensitive gamma analysis with local (L) dose**–**error normalization, *γ* (2%L/2 mm). It is clear that for the larger fields the beam profiles disagree somewhat, particularly with increasing depth. For the 10 MVFFF beam, the Calculator profile and Pinnacle straddled the experimental curve, although the Calculator was closer. On the other hand, Pinnacle showed better agreement with the measurement for 15 MV. The disagreement for the MLC‐defined 3 × 3 cm^2^ 15 MV field inside the 10 × 10 cm^2^ jaw opening is due entirely to the 2.9% difference in computed outputs for a partially obscured distributed secondary source (all comparisons were done in absolute dose mode). Pinnacle and Calculator calculation results straddled our experimental output value, with Pinnacle being 0.7% high and the Calculator 2.2% low, both reasonable values. The Pinnacle dose matched exactly the median of measured values from Kerns et al.^22^ Scaling the Calculator dose accordingly lead to a 100% agreement at the 2%L/2 mm level beyond the d_max_.

### 3.B Slab inhomogeneities

**Figure 2:**
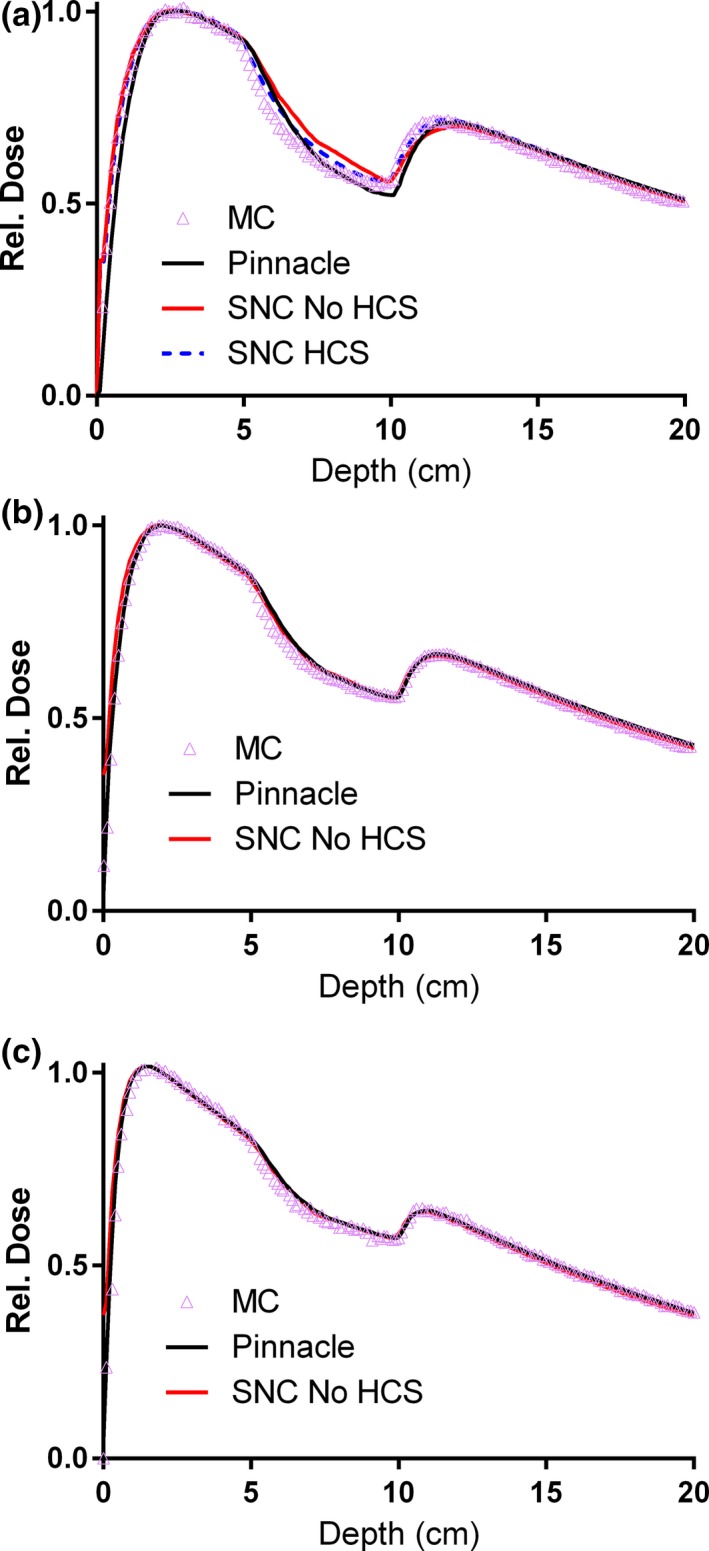
Central axis PDDs on the lung slab phantom for the 2 × 2 cm^2^ field: 15MV (a), 10 MVFFF (b), and 6MV (c).

The central axis PDDs on the lung slab phantom for a small (2 × 2 cm^2^) field are shown in Fig. 2. For the 15 MV beam (Fig. 2(a)) one can see the difference in lung dose between Pinnacle, Non‐HCS Calculator, and HCS‐corrected Calculator. The HCS correction improved agreement with Pinnacle and MC in the simple geometry, as reported previously.^27^ For the lower energies (Figs. 2(b) and2(c)), the difference between Pinnacle and the standard Calculator mode in the inhomogeneity was minimal (< 1.3% and 0.7% of D_max_ for 10 MVFFF and 6 MV, respectively), and both were sufficiently close to MC. Therefore, the effect of the HCS correction was not further studied for those energies. The larger fields (not shown) exhibited the same trends but to a smaller degree.

### 3.C The Calculator vs. point dose measurements

For the point doses in the homogeneous phantoms or portions thereof (e.g., excluding the target location in the Thoracic phantom), the mean Calculator vs. the IC difference was −0.3 ± 0.8% (1 SD). The range was from −2.0 to 1.0%. For the plans based on the Guideline 5a Lung case as delivered to the Thorax phantom, the 15MV IMRT and VMAT plans showed 2.8 and 4.0% difference from the IC in the target respectively. Pinnacle doses at the same point were within 1.4% of the IC. With the HCS correction applied, the disagreement was reduced to −0.7% and 1.6% for the 15 MV IMRT and VMAT plans, respectively, while the change in the homogeneous portion of the phantom was minimal. The findings for the Lung plans with both lower energies were unremarkable at both measurement points in the Thorax phantom.

### 3.D The Calculator vs. biplanar diode array (homogeneous phantom)

**Figure 3:**
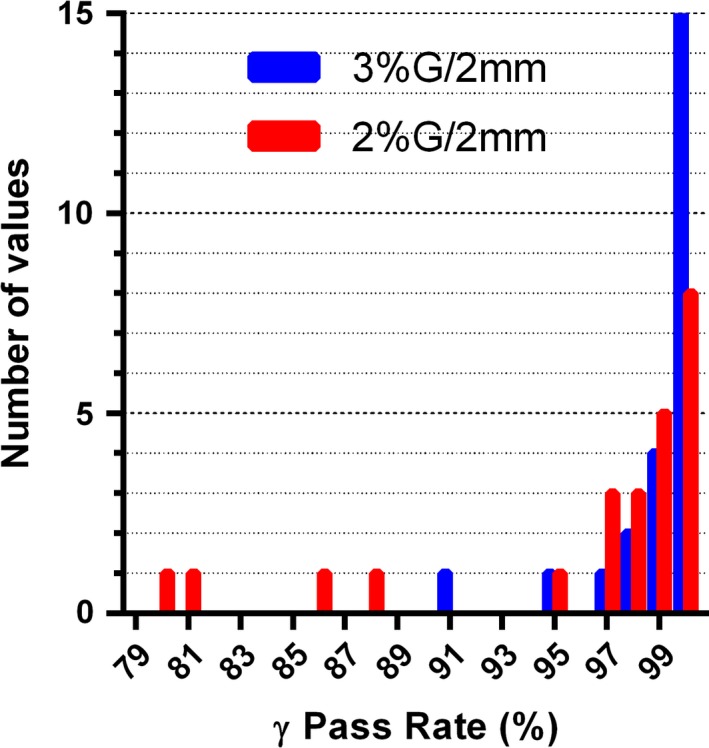
Frequency distribution of the *γ*‐analysis passing rates comparing the Calculator to the Delta4 for all Guideline 5a test cases and energies. N = 24.

The average *γ* (3%G/2 mm) and *γ* (2%G/2 mm) passing rates of the Calculator against the Delta^4^ measurements were 98.9 ± 2.1% and 96.1 ± 6.4% respectively. The Calculator produced *γ* (3%G/2 mm) agreement rates with the Delta^4^ measurements above 95% in all the studied cases but two, and all were above 90% (Fig. 3). Both lower passing rates are associated with the 6 MV Anal case plans (94.8% for VMAT and 91.4% for IMRT) (Fig. 3)

### 3.E The Calculator vs. 3D measurement‐guided dose reconstruction (not including lung)

**Figure 4:**
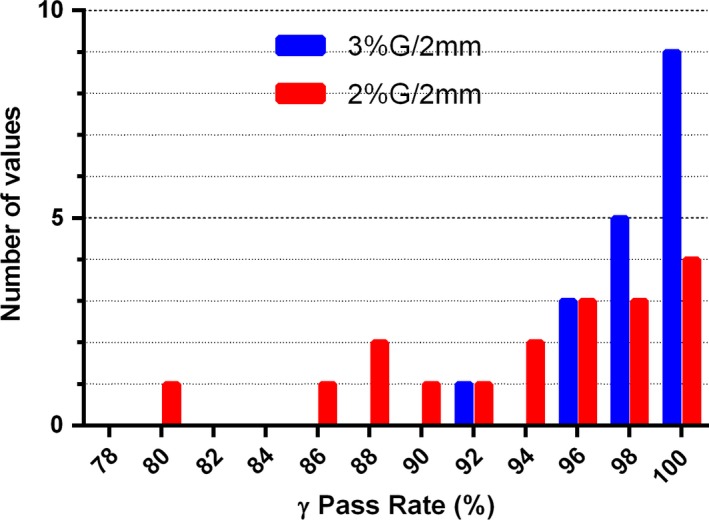
Frequency distribution of the *γ*‐analysis passing rates comparing the Calculator to ACPDP measurement‐guided dose reconstruction on the patient datasets for Guideline 5a test cases excluding the Lung (all energies). N = 18.

**Figure 5:**
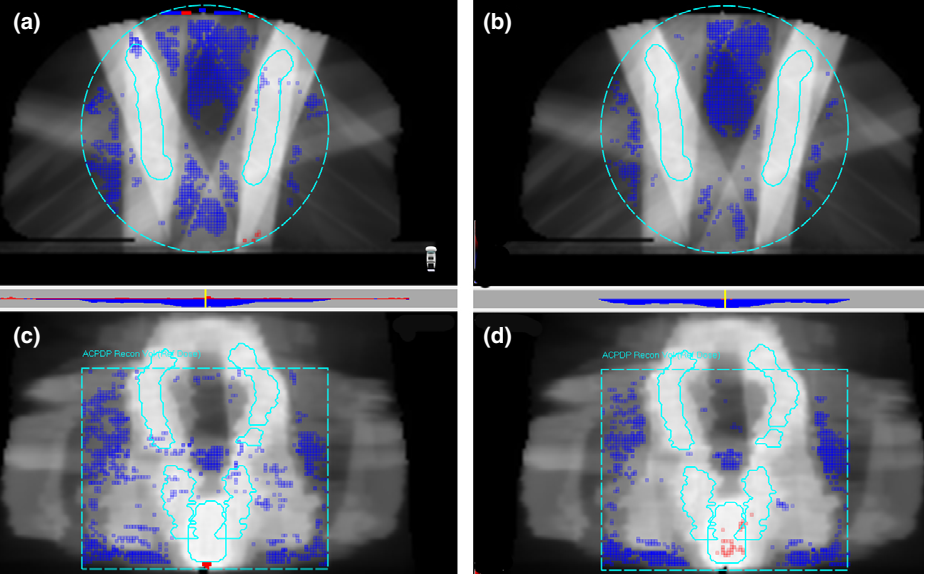
Graphical representation of the *γ*(2%G/2 mm) comparison for the 6X Anal IMRT plan between the Calculator and ACPDP (left column) and Pinnacle vs. ACPDP (right column). The highlighted pixels where the gamma analysis fails are overlaid on the dose map. The targets are also shown. The transverse cuts (a), (b) are taken superiorly to demonstrate the areas of failure between the irradiated nodal chains. The coronal cuts (c), (d) are 2 cm posterior to the midline, where both the primary and secondary targets are prominently present.

In the next step, the agreement between the measurement‐guided dose reconstruction on the patient datasets using the ACPDP method, and the Calculator followed the same trend as the direct comparison on the homogeneous phantom (compare Figs. 4 and 3). The Guideline 5a Lung plans were excluded from the comparison. The average *γ* (3%G/2 mm) and *γ* (2%G/2 mm) passing rate were 98.2 ± 2.0%. and 93.8 ± 5.7% respectively. The only test case falling below the *γ* (3%G/2 mm) 95% agreement level (91.8%) was again the 6 MV IMRT Anal plan. To clearly demonstrate the predominant areas of failure, representative transverse and coronal cross‐sections for that plan are shown in Fig. 5 A and D, with the highlighted pixels representing the areas of 2%G/2 mm analysis failure. The failing pixels are largely concentrated in the lower dose areas peripheral to, or in between the targets. The rest of the ACPDP doses for both VMAT and IMRT plans agreed with the Calculator for 96.4% of the points or better (3%G/2mm).

### 3.F The Calculator vs. primary TPS on the patient CT (not including lung)

**Figure 6:**
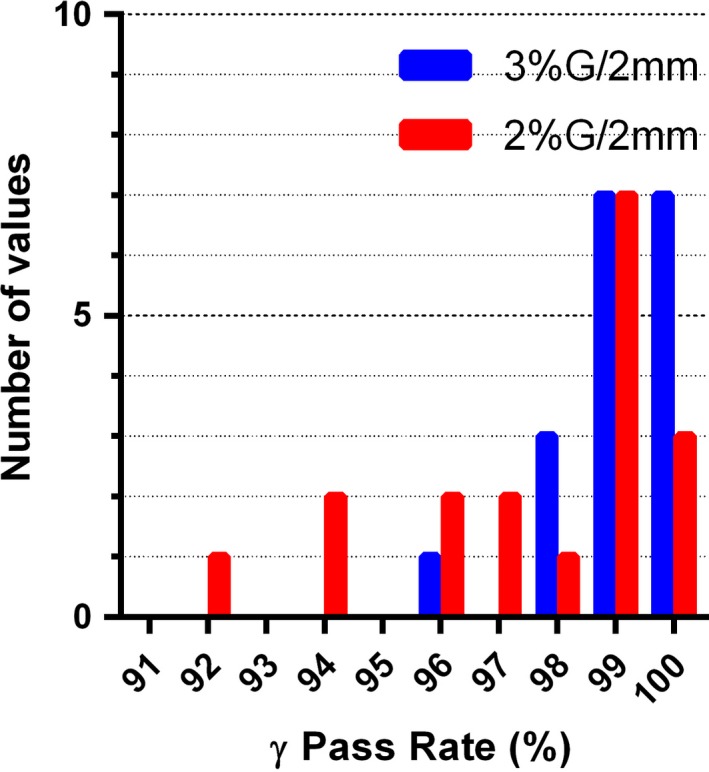
Frequency distribution of the *γ*‐analysis passing rates comparing the Calculator to Pinnacle on the patient datasets for Guideline 5a test cases, excluding the Lung (all energies). N = 18.

The mean *γ* (3%G/2 mm) and *γ* (2%G/2 mm) passing rates comparing the Calculator to Pinnacle were 99.0 ± 1.0% and 97.5 ± 2.4%, respectively. The corresponding ranges are 96.3 to 100% and 91.6 to 99.7% respectively. The frequency distributions are shown in Fig. 6. The three instances with the *γ*(2%G/2mm) passing rates below 95% are all associated with higher energies on the H&N dataset, which is unlikely to be seen in practice. Of note, the patterns of *γ* (2%G/2 mm) analysis failure against ACPDP for the 6 MV IMRT Anal plan are visually similar between the Calculator and ACPDP (Fig. 5). Not surprisingly the 2%G/2 mm gamma passing rate between the Calculator and TPS is 99.1%.

### 3.G The calculator vs. primary TPS (lung)

The agreement level between the Calculator and Pinnacle for the Lung plan is energy‐, calculation mode‐, and dataset‐dependent, which necessitates a more detailed discussion. As can be seen in Table 1, passing rates on the Guideline 5a Lung dataset decrease as the beam energy increases, which is particularly clear with the 2% dose**–**error threshold. The HCS correction applied to the 15 MV plans did not lead to an improvement. The data for the same plans recalculated on the Thorax phantom are presented in Table 2. Unlike with the original dataset, the improvement in agreement with Pinnacle due to the HCS correction is substantial. However, that by itself does not prove that the HCS correction leads to more accurate results. A comparison with a definitive standard, such as an MC calculation, is necessary. Such analysis was performed for a 5‐beam 15 MV 3D plan. Pinnacle and the Calculator were in agreement by the *γ* (3%G/2 mm) analyses for 98.5% (no HCS) and 97.6% (with HCS) of the voxels. Subsequent comparisons with MC are presented in Table 3. The Calculator without the HCS correction showed the best overall agreement with MC by gamma analysis, as well as the target dose‐volume histogram (DVH) agreement. On the other hand, the Pinnacle lung DVHs were the closest to MC.

**Table 1:** Gamma analysis passing rates comparing the Calculator to Pinnacle on the Guideline 5a Lung dataset

Energy/plan	*γ*‐Analysis passing rate (%)			
	Non‐HCS		HCS	
	2%G/2 mm	3%G/2 mm	2%G/ 2mm	3%G/ 2mm
6 MV‐VMAT	99.4	100.0		
6 MV‐IMRT	98.6	99.8		
10 MVFFF‐VMAT	93.9	98.9		
10 MVFFF‐IMRT	94.2	98.6		
15 MV‐VMAT	82.9	93.4	82.8	92.4
15 MV‐IMRT	89.9	96.3	89.0	95.5

**Table 2:** Gamma analysis passing rates comparing the Calculator to Pinnacle for the same plans as in Table 1 but recalculated on the Thoracic phantom

Energy/plan	*γ*‐Analysis passing rate (%)			
	Non‐HCS		HCS	
	2%G/2 mm	3%G/2 mm	2%G/2 mm	3%G/2 mm
6 MV‐VMAT	99.6	100.0		
6 MV‐IMRT	98.8	99.8		
10 MVFFF‐VMAT	92.0	98.6		
10 MVFFF‐IMRT	90.0	97.7		
15 MV‐VMAT	76.4	86.1	90.8	97.2
15 MV‐IMRT	81.9	89.6	90.9	95.8

**Table 3:** Gamma analysis passing rates comparing MC to Pinnacle and the Calculator (with and without the HCS correction) for the 15 MV 3D plan on the Thoracic phantom

*γ*‐Analysis passing rate (%)					
MC vs. Pinnacle		MC vs. calculator (no HCS)		MC vs. calculator (HCS)	
2%G/2 mm	3%G/2 mm	2%G/2 mm	3%G/2 mm	2%G/2 mm	3%G/2 mm
87.1	92.1	95.7	97.4	92.1	94.9

## 4 Discussion

The calculator was put through a series of tests representing a subset of those required for commissioning of a primary TPS dose engine.^23^, ^38^ The mean IC point dose error in the homogeneous phantoms is well below the 1.5% expectation.^23^, ^39^ According to the forthcoming recommendation on IMRT QA criteria, 95% of the points on a homogeneous phantom passing the 3%G/2 mm/10% gamma analysis should constitute the tolerance limit, with the action limit set at 90% (private communication). While no compelling data exist to suggest that 3%/2 mm criteria hold an advantage in sensitivity/specificity over 3%/3 mm, reducing the distance‐to‐agreement tolerance to 2 mm appears intuitive, given that recommended distance‐type tolerances in TG‐142^40^ for IMRT machines are 1 to 2 mm.

Comparisons between the Calculator and direct measurements by the diode array indicated that the Calculator performed at the accuracy level that could be expected for routine patient‐specific QA from a primary TPS. Similar results were seen for the volumetric comparisons on the Guideline 5a patient datasets (excluding the Lung) with measurement‐guided dose reconstruction. In both series of experiments only some Anal plans exhibited less than 95% (but above 90%) passing rates at the 3%G/2 mm level. This is one of the most challenging plan classes, featuring large bifurcating targets that require a rather high number of segments, thus challenging the dose engine model accuracy under the MLC leaves and in the penumbra. As such, these types of cases often exhibit the largest dosimetric discrepancies regardless of the dose calculation algorithm. The 6 MV Anal IMRT plan's pattern of pixels failing the more stringent 2%G/2 mm gamma analysis against ACPDP (Fig. 5) is similar between the Calculator and TPS. The most challenging are the low dose areas outside and in particular in between the targets, indicating imperfect dose calculations at the voxels spending a relatively large proportion of time under the closed MLC leaves. A qualitatively similar pattern of failure is observed with the Delta^4^, increasing the likelihood that the observed errors are real rather than a measurement artifact, as a 2% dose**–**error threshold is pushing the accuracy of diode arrays. Overall, considering a generic beam model used in the Calculator, the demonstrated level of agreement on the homogeneous or mildly inhomogeneous datasets should be considered satisfactory.

The situation is more complicated in the thoracic region. The slab geometry model indicates that for the lower energies (6 and 10 MV), the difference between Pinnacle and the Calculator PDDs at the tissue/lung interfaces is minimal and both are close to MC. However, the differences were noticed for 15 MV, with the HCS‐corrected Calculator PDD being closer to Pinnacle and MC than the uncorrected data. But that did not translate into a better overall agreement between Pinnacle and the Calculator on the Guideline 5a Lung dataset for modulated plans (93–96% for *γ* (3%G/2 mm)). The plans were then projected on the Thorax phantom, which contains better defined, sharp interfaces between 0.21 g/cm^3^ and ~1 g/cm^3^ densities. In that configuration, the HCS‐corrected Calculator showed substantial improvement in agreement with Pinnacle for the modulated plans (Table 2). While Pinnacle was widely tested in lung, it cannot be considered the standard and in fact has been shown to be rather inaccurate in certain situations.^34^ Unfortunately, we did not have the ability to recalculate the modulated plans with MC. However, a five‐field 15 MV 3D plan was compared with MC on the Thorax phantom. The best overall agreement with MC in this case was observed for the standard (no HCS) Calculator dose. The nature of the HCS correction – changing the CT dataset densities ‐ is nonphysical. It was optimized for slab inhomogeneities but does not improve accuracy in all situations. We therefore cannot recommend its routine use without further investigation. On balance, this is largely an academic point. The correction makes the most difference for the SBRT‐type lung treatments, the precise situation where the use of energies above 10 MV should be rather limited.

There is no guidance document yet on IMRT dose distribution verification by independent calculation, with or without some empirical input. In the meantime, it seems reasonable to apply the forthcoming experimental verification criteria in most situations. However, there are additional uncertainties related to calculating dose in lung, and appropriate criteria need to be developed, perhaps such as the varying action limits based on the algorithm similarity and case complexity established for secondary checks in non‐IMRT radiotherapy.^2^

Finally, we must point out that subtle dose differences could stem from the way the different algorithms calculate or report dose to different tissue types.^41^ For example, both Pinnacle and the Calculator determine the tissue type from their CT to density conversion tables, using the mass and electron densities, respectively. This tissue assignment is then used to look up the appropriate mass energy absorption coefficient for TERMA calculations. The Pinnacle assignment table does not include water, while in the Calculator CT voxels corresponding to approximately unit density would resolve to water. This difference in dose reporting could potentially lead to up to ~1% error even on an ideal unit‐density phantom, and is hard to control for a patient CT dataset.

## 5 Conclusions

We benchmarked a fast GPU‐based independent S/C dose engine that produces 3D dose distributions on the patient CT datasets for comparison with the TPS IMRT/VMAT calculations. Comparisons with the ion chamber, diode array measurements, and measurement‐guided 3D dose reconstruction for challenging datasets reveal the accuracy level expected in routine patient‐specific testing [≥ 95% *γ* (3%G/2 mm) passing rates in most cases]. Direct comparison with the Pinnacle TPS on the realistic CT datasets showed similar agreement. The alternative additional heterogeneity correction can change the dose noticeably in some situations with the higher energy beam (15 MV). However, this correction does not always result in the more accurate dose calculation and should be used with caution.

## Conflict of Interest

JK and RH are employees of SNC. No other conflict of interest is reported.
